# Expression of a cucumber *alanine aminotransferase2* gene improves nitrogen use efficiency in transgenic rice

**DOI:** 10.1186/s43141-019-0010-7

**Published:** 2019-11-12

**Authors:** Atmitri Sisharmini, Aniversari Apriana, Nurul Khumaida, Kurniawan Rudi Trijatmiko, Bambang Sapta Purwoko

**Affiliations:** 1 Plant Breeding and Biotechnology Study Program, Departement of Agronomy and Horticulture, IPB University (Bogor Agricultural University), Jl. Meranti, Kampus IPB Darmaga, Bogor, 16680 Indonesia; 2 Indonesian Center for Agricultural Biotechnology and Genetic Resources Research and Development, Jl. Tentara Pelajar 3A, Bogor, 16111 Indonesia

**Keywords:** *Alanine aminotransferase*, Cucumber, Nitrogen use efficiency, Rice

## Abstract

**Background:**

Rice can absorb less than 40% of applied nitrogen fertilizer, whereas the unabsorbed nitrogen fertilizer may cause environmental problems, such as algal blooms in freshwater and increased production of nitrous oxide, a greenhouse gas which is 300 times more potent than carbon dioxide. Development of nitrogen use efficient (NUE) rice is essential for more environmentally friendly rice production. Recently, NUE rice has been developed by root-specific expression of *alanine aminotransferase* (*AlaAT*) gene from barley, a monocot plant. Therefore, we tested the efficacy of *AlaAT* gene from cucumber in transgenic rice, aiming to provide evidence for the conservation of *AlaAT* gene function in monocot and dicot.

**Results:**

*AlaAT* gene from cucumber (*CsAlaAT2*) has been successfully cloned and constructed on pCAMBIA1300 plant expression vectors under the control of tissue-specific promoter *OsAnt1*. *Agrobacterium tumefaciens*-mediated transformation of Indonesian rice cv. Fatmawati using this construct produced 14 transgenic events. Pre-screening of T1 seedlings grown in the agar medium containing low nitrogen concentration identified selected events that were superior in the root dry weight. Southern hybridization confirmed the integration of T-DNA in the selected event genomes, each of them carried 1, 2, or 3 T-DNA insertions. Efficacy assay of three lead events in the greenhouse showed that in general transgenic events had increased biomass, tiller number, nitrogen content, and grain yield compared to WT. One event, i.e., FAM13, showed an increase in yield as much as 27.9% and higher plant biomass as much as 27.4% compared to WT under the low nitrogen condition. The lead events also showed higher absorption NUE, agronomical NUE, and grain NUE as compared to WT under the low nitrogen condition.

**Conclusions:**

The results of this study showed that root-specific expression of cucumber *alanine aminotransferase2* gene improved nitrogen use efficiency in transgenic rice, which indicate the conservation of function of this gene in monocot and dicot.

## Background

Effort to increase rice production in the world must continue to meet the increase in rice demand. The green revolution in 1960–1970 has achieved remarkable increase in rice production. However, the excessive use of fertilizers and pesticides has negative impacts on the environment and soil fertility. The green revolution and the doubling of food production over the last four decades are associated with an increase in the use of nitrogen fertilizers [10]. The use of nitrogen also rises from 3.5 million tonnes in 1960 to 87 million tonnes in 2000 and is expected to increase to 249 million tonnes by 2050 [32]. The current fertilizer dose of 400–600 kg urea/ha commonly used by farmers in Indonesia exceeds the government’s recommendation of 200–260 kg/Ha [33].

Nitrogen is actively absorbed by plants from the soil in the form of ammonium and nitrate [2, 8, 9, 21, 25]. Nitrogen is an important nutrient used by plants for their growth and development as components of DNA, proteins, enzymes, and metabolite products involved in synthesis and transfer of energy [6, 17]. Nitrates and ammonium are the two inorganic nitrogen compounds existing in agricultural soil and very mobile in the soil. The plant is only capable of using about 30–40% of the available nitrogen. Thus, more than 60% of soil nitrogen is lost through a combination of leaching, surface run-off, denitrification, volatilization, and soil microbial consumption. When calculated, 1% increase in nitrogen use efficiency can save 1.1 billion US dollars per year [15]. Therefore, minimizing N losses can reduce environmental pollution and reduce production cost.

Among the genes that have been used to improve nitrogen use efficiency in plant is *alanine aminotransferase* (*AlaAT*) [5, 28]. *AlaAT* is a pyridoxal-5′-phosphate-dependent enzyme found in all parts of the plant. This enzyme activity is not only found in leaves and roots, but also in other tissues such as the endosperm and flower [13]. The pattern of expression of *AlaAT* shows that this enzyme is involved in important biochemical reactions throughout the plant life cycle. This enzyme catalyzes reversible reaction between pyruvate and glutamate to alanine and 2-oxoglutarat, connecting metabolism of carbon with synthesis of various amino acids [20, 26].

Research on the use of *AlaAT* gene from barley has been reported on *Brassica napus* using btg26 root-specific promoter. Brassica’s transgenic plants show an improvement in the nitrogen use efficiency compared to non-transgenic, as indicated by an increase in biomass and seed yield in low N condition at laboratory and field tests [5]. Heterologous expression of barley *AlaAT* in transgenic rice has also been conducted using a specific tissue promoter *OsAnt1*. This modification can increase biomass and grain yield significantly in transgenic rice compared to control plants when the plants are supplied with sufficient nitrogen. Transgenic plants also showed changes in nitrogen content and metabolism significantly. This result indicates the improvement in nitrogen use efficiency [28].

Improvement of N uptake in cereal plants can be done by manipulating the downstream step in the nitrogen metabolism pathway [28]. The development of food crops that can take and process N for assimilation more efficiently will make efficient use of N fertilizer, which can reduce production cost and environmentally friendly [7]. The extensive study on the *AlaAT* gene to improve nitrogen use efficiency has used the gene from barley that belongs to monocots. Study on the dicots *AlaAT* genes needs to be initiated to investigate the possible functional conservation of this gene among monocots and dicots and to find orthologous that might be useful for gene optimization purpose in the future.

The objective of this study was to investigate the efficacy of cucumber *AlaAT2* gene driven by *OsAnt1* root-specific promoter in improving the nitrogen use efficiency in transgenic rice. Our results indicate that the cucumber *AlaAT2* gene can be used for the development of transgenic rice varieties that are efficient in using nitrogen fertilizers.

## Methods

### Generation of plant transformation constructs and transgenic rice

Fragment encompassing the full-length coding region of *CsAlaAT2* was amplified from F1 hybrid cucumber (*Cucumis sativus L*) cv. Roberto (Chia Tai Seeds Co.) root cDNA. Since the activity of *alanine aminotransferase* enzyme is induced under hypoxia conditions [4], plant samples were subjected to hypoxia conditions prior to RNA isolation following the procedure described by Kendziorek et al. [16]. Briefly, hypoxia was imposed by submerging roots and shoots of 10-day-old cucumber seedlings up to 1/3 length in flasks containing Yoshida solution covered by about 2-cm layer of oil to prevent gas exchange. Total RNA was isolated from hypoxic-treated root using RNeasy Plant Mini Kit as described by the supplier (Qiagen, Germantown, MD, USA). Amplification of Cs*AlaAT2* gene was performed using a blend of FastStart *Taq* DNA Polymerase (Roche) and KAPA Hifi DNA Polymerase Hotstart (Kapa Biosystems, Wilmington, MA, USA) and oligonucleotides *CsAlaAT2*-FL-F (5′-gcggatccCGGCTACACCACCAACTCTT-3′) and *CsAlaAT2*-FL-R (5′-gcggtaccTGCACCTTTGATACGCAGGA-3′). The oligonucleotides introduced *Bam*HI and *Kpn*I restriction sites (lowercase) to the amplified fragments at their 5′ and 3′, respectively. *OsAnt1* promoter was amplified from genomic DNA of rice cv. Kitaake using FastStart *Taq* DNA Polymerase and specific oligonucleotides for *OsAnt1* promoter [35]. The oligonucleotides introduced *Hin*dIII and *Bam*HI restriction sites to the amplified fragments at their 5′ and 3′, respectively. *CsAlaAT2* and *OsAnt1* fragments were introduced to the pGEM-T Easy vector as described by the manufacturer (Promega, Madison, WI) and subsequently sequenced before digestion and ligation to the binary vector. Promoter and gene fragments with appropriate compatible cohesive ends were ligated together to the *Hin*dII-*Kpn*I digested pCAMBIA1300int-prGluA2-GUS-tNOS binary vector [34] as a substitute for GluA2 promoter and GUS gene to create pCAMBIA1300int-prOsAnt1-*CsAlaAT*2-tNOS. The binary vector was transformed into *Agrobacterium tumefaciens* strain LBA4404 [11] using freeze-thaw method [12]. Transformation of rice cv. Fatmawati (collection of Indonesian Center for Rice Research with accession no. 4825) was performed following the method described by Slamet-Loedin et al. [30]. Briefly, immature embryos were co-cultivated with *Agrobacterium* suspension for 7 days. The elongated shoots were removed, and the embryos were transferred to resting medium. After 5 days, the embryos were transferred to selection medium containing hygromycin and incubated for 10 days. The second selection was performed for 10 days. Embryogenic calli were selected and transferred to fresh selection medium. After 10 days, the resistant calli were transferred to pre-regeneration medium and incubated for 10 days. Proliferating calli with green spots were transferred to regeneration medium. Plantlets were transferred to rooting medium and incubated for 14 days. Direct PCR with leaf disc as template was performed to confirm the presence of *CsAlaAT2* transgene in transformed plants using KAPA 3G Plant PCR Kits (Kapa Biosystems) and oligonucleotides *CsAlaAT*2-F: 5′-ATAAAGCAGAAGGCGCAATG-3′ and tNOS-R: 5′-ATTGCCAAATGTTTGAACGA-3′.

### Pre-screening of events on agar medium containing low nitrogen concentration

Pre-screening of events was conducted by germinating T1 seeds of transgenic rice events on agar medium containing macronutrients, micronutrients, and vitamins following Murashige and Skoog [23], except for NH_4_NO_3_ and KNO_3_ where 1/16 strength was used. Each T1 seed was germinated in a glass test tube (25-mm diameter × 200-mm height) containing 25-mL agar medium. The seedlings were incubated under continuous light at 28 °C. The seedlings were harvested at 21 days after seeding for evaluation of 6 traits: (1) root length, (2) shoot length, (3) root wet weight, (4) shoot wet weight, (5) root dry weight, and (6) shoot dry weight. The root dry weight was used as the criterion to select the lead events because previous studies show that expression of barley alanine aminotransferase leads to early establishment of a vigorous root system in transgenic rice [27, 28].

### Molecular characterization

**Fig. 1:**
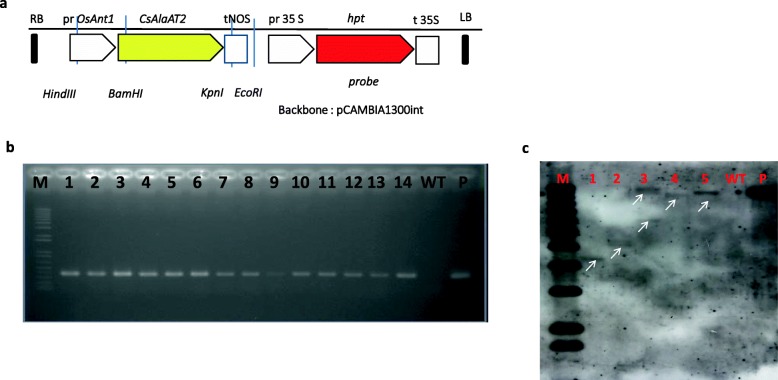
Development and molecular characterization of *CsAlaAT2* transgenic rice event. **a** Schematic diagram of the T-DNA construct *OsAnt1::CsAlaAT2*, containing the *CsAlaAT2* gene with the NOS terminator under the control of the *OsAnt1* promoter. RB, right border; pr*OsAnt1*, promoter antiquitin1; *CsAlaAT2*, *Cucumis sativus Alanine aminotransferase*; tNOS, terminator Nopaline synthase; pr 35 S, promoter 35 S; hpt, hygromycin phosphotransferase; C35S, cauliflower 35 S; LB, left border. **b** PCR amplification of cucumber *AlaAT* on T0 transgenic rice lines. M = 1 kb DNA ladder plus; 1–14 = transgenic lines, WT = wild type, P = plasmid control. **c** Southern blot analysis showing the copy number of *hpt* gene insertion in the T0 generation of transgenic lines. Hpt gene insertion is shown by white arrow. M = marker 1 kb ladder, WT = wild type, 1 = FAM3, 2 = FAM4, 3 = FAM5, 4 = FAM 9, 5 = FAM13, P = plasmid of pCAMBIA1301-*CsAlaAT2*

Five events showing higher root dry weight than control (Table 1) were estimated for the T-DNA insertion number using Southern blot analysis following the method described by Trijatmiko et al. [35]. Briefly, DNA samples were digested with a single-cutter enzyme (*EcoRI*), separated by agarose gel electrophoresis, denatured, blotted to a nylon membrane, and hybridized with a hygromycin phosphotransferase (*hpt*) probe (Fig. 1a). Probe was labeled with DIG-dUTP (Roche), by the method of polymerase chain reaction using oligonucleotides *hpt*-F (5′-GCATCTCCCGCCGTGCAC-3′) and *hpt*-R (5′-GATGCCTCCGCTCGAAGTAGCG-3′). Detection was conducted using anti-digoxigenin antibody conjugated to alkaline phosphatase (Roche) and a chemiluminescence substrate (Roche). The light signal was captured on X-ray film (Hyperfilm ECL, GE Healthcare, Chicago, WI, USA).

**Table 1:** Root and shoot characters of control and transgenic rice plants in MS medium containing 1/16 concentration of nitrogen

Line	Root length (cm)	Shoot length (cm)	Wet biomass		Dry biomass	
			Root (g)	Shoot (g)	Root (g)	Shoot (g)
Control	11.74	33.32	0.11	0.16	0.019	0.031
FAM1	9.47	31.40	0.11	0.16	0.018	0.027
FAM2	11.17	32,90	0.11	0.17	0.019	0.032
FAM3	9.10	33.07	0.12	0.17	0.020	0.028
FAM4	9.60	32.13	0.12	0.17	0.021	0.034
FAM5	16.37*	32.43	0.17**	0.19	0.033*	0.033
FAM6	11.33	32.53	0.11	0.17	0.017	0.025
FAM8	8.30	22.87	0.09	0.13	0.016	0.019
FAM9	10.83	33.60	0.11	0.16	0.018	0.030
FAM10	14.07	33.13	0.10	0.17	0.014	0.027
FAM12	11.40	29.30	0.08	0.14	0.014	0.022
FAM13	9.80	28.67	0.19**	0.18	0.030*	0.033
FAM16	9.87	32	0.093	0.183	0.016	0.025
FAM17	9.97	32.43	0.107	0.180	0.017	0.026

*Significantly higher than control at *P* < 0.05

**Significantly higher than control at *P* < 0.01

### Phenotypic evaluation of transgenic plant

T1 seeds of three selected events, i.e., FAM3, FAM5, and FAM13, were used for phenotypic evaluation in the greenhouse. Seeds from control plants and transgenic lines were germinated in petri dishes for 2 days and subsequently transferred to germination trays. PCR was conducted on 2-week-old seedlings to select transgene-positive plants. The selected seedlings were transferred to pots for evaluation of nitrogen use efficiency.

Evaluation was conducted in a greenhouse with applied nitrogen treatments following Selvaraj et al. [27], i.e., N0 = 0 kg/ha, N1 = 90 kg/ha, N2 = 180 kg/ha, which was converted to the volume of soil in the pot. For N1 and N2, nitrogen was applied in three equal splits at 2 days after transfer (DAT), 10 DAT, and 30 DAT.

Observations were conducted on agronomic characters, including plant height, number of panicle, panicle length, number of filled grain, percent seed set, 1000-grain weight, yield, root dry weight, and shoot dry weight. Root nitrogen concentration and shoot nitrogen concentration were measured by Kjeldahl method [14].

The agronomic data were analyzed by one-way ANOVA implemented in the Statistical Analysis System (SAS Institute Inc., Cary, NC, USA). In the presence of a significant effect for the model source of variation, means separation procedure was used with Fisher’s least significant difference at the level of *P* = 0.05. The value of absorption NUE (aNUE) was calculated by dividing the total N absorbed by the plant (gram) by the amount of N applied (gram). Agronomic NUE (agNUE) was calculated by dividing the total dry biomass of plant (gram) by the amount of N applied (gram). Grain NUE (gNUE) was calculated by dividing the yield of plant (gram) by the amount of N applied (gram) [19, 22].

## Results

### Generation of plant transformation constructs and transgenic rice

PCR product encompassing the open reading frame of *CsAlaAT2* gene (∼ 1.565 kb) has been successfully amplified from cucumber root cDNA, inserted into the pGEM-T easy vector system, and sequenced. The *CsAlaAT2* fragment from a correct clone was excised with *Bam*HI and *Kpn*I and ligated together with a *Hin*dIII- and *Bam*HI-digested pOsAnt1 fragment into a binary vector pCAMBIA1300int-tNos to produce a plant transformation vector pCAMBIA1300int-*pOsAn1t*-*CsAlaAT2-*tNos (Fig. 1a). Subsequently, the vector was transformed into the *Agrobacterium tumefaciens* strain LBA4404 and used for rice transformation. The number of transgenic events created with pCAMBIA1300int-*pOsAnt*-*CsAlaAT2*-tNos was 14. Confirmation by PCR amplification showed that all 14 events carried *CsAlaAT2* transgene (Fig. 1b). Of the positive events, 13 produced at least 200 seeds.

### Pre-screening of events on agar medium containing low nitrogen concentration

T1 plants of 13 selected transgenic rice events (FAMs) were pre-screened on agar medium with low nitrogen. Observation on the 21 days after planting showed that root biomass of 2 transgenic events, i.e., FAM5 and FAM13, were significantly higher than control plant (Table 1).

### Molecular characterization of selected events

Southern blot analysis using *Eco*RI that cuts only one time in the T-DNA and *hpt* probe was conducted on 5 selected transgenic T0 events, i.e., FAM3, FAM4, FAM5, FAM9, and FAM13, and wild type. Of the selected events, 3 events (FAM3, FAM9, and FAM13) showed single insertion of T-DNA, 1 event (FAM4) showed 2 insertions of T-DNA, and 1 event (FAM5) showed 3 insertions of T-DNA. As expected, wild-type plant (control) did not show any hybridization (Fig. 1c). The three events with single insertion of T-DNA showed different sizes of bands, indicating that each of them is a unique event.

### Phenotypic evaluation of selected events

Phenotypic evaluation of three selected events, i.e., FAM3, FAM5, and FAM13, was conducted in the greenhouse. T1 plants of selected events and wild type were grown in the pots under 3 different N dosages, i.e., 0 kg/ha, 90 kg/ha, and 180 kg/ha.

The correlation coefficients between traits were calculated and are presented in Table 2. Tiller number was the only trait that showed a positive correlation with grain yield. Tiller number had a positive correlation with root dry weight. Shoot dry weight had positive correlations with root dry weight, root N concentration, and shoot N concentration.

**Table 2:** Yield parameters measured from greenhouse experiment of three *CsAlaAT2* lead events and WT under three N fertilizer regimes

N level	Genotype	RDW	SDW	RNC	SNC	TN	GY
N 0%	Control (WT)	4.57^e^	18.50^f^	0.06	0.14^c^	7.0^bc^	30.59^bc^
	FAM3	6.90^bcd^	19.95^def^	0.10	0.22^bc^	8.3^ab^	29.49^bc^
	FAM5	8.46^ab^	23.13^cdef^	0.12	0.34^ab^	8.0^abc^	29.11^bc^
	FAM13	5.33^cde^	19.44^ef^	0.06	0.23^bc^	9.0^a^	34.32^abc^
N 50%	Control (WT)	4.92^de^	20.84^cdef^	0.08	0.20^bc^	6.3^bc^	31.48^bc^
	FAM3	7.49^bc^	24.11^bcd^	0.14	0.25^bc^	8.3^ab^	37.58^ab^
	FAM5	7.38^bcd^	20.36^cdef^	0.13	0.30^abc^	8.7^ab^	32.62^abc^
	FAM13	7.09^bcd^	24.69^bcd^	0.08	0.24^abc^	9.0^a^	40.28^a^
N 100%	Control (WT)	5.32^cde^	24.51^bcde^	0.09	0.18^bc^	7.0^bc^	30.55^bc^
	FAM3	9.30^ab^	34.83^a^	0.13	0.42^a^	8.3^ab^	33.02^abc^
	FAM5	7.28^bcd^	25.38^bc^	0.09	0.27^abc^	9.0^a^	28.78^c^
	FAM13	10.13^a^	29.27^b^	0.12	0.23^bc^	9.0^a^	34.09^abc^
N level (N)		*	**	ns	ns	ns	*
Genotype (G)		*	*	ns	*	**	*
N × G		*	*	ns	ns	ns	ns
CV (%)		18.59	11.37	34.31	35.50	11.08	13.32

Means with the same letter are not significantly different

*Significant at *P* < 0.05

**Significant at *P* < 0.01

Significant differences were detected in N treatment (N), genotype (G), and N × G interaction for root dry weight and shoot dry weight (Table 3). Significant differences were also detected in genotype (G) for shoot N concentration, tiller number, and grain yield (Table 3).

**Table 3:** Correlation among traits (all correlations shown are significant at *P* < 0.05)

Trait	PH	TN	PL	GPP	RDW	SDW	RNC	SNC
TN	–							
PL	–	–						
GPP	–	–	–					
RDW	–	0.356	–	–				
SDW	–	–	–	–	0.636**			
RNC	–	–	–	–	0.553**	0.368		
SNC	–	–	–	–	0.397	0.554**		
GY	–	0.449**	–	–	–	–	–	–

** Significant at *P* < 0.001

**Fig. 2:**
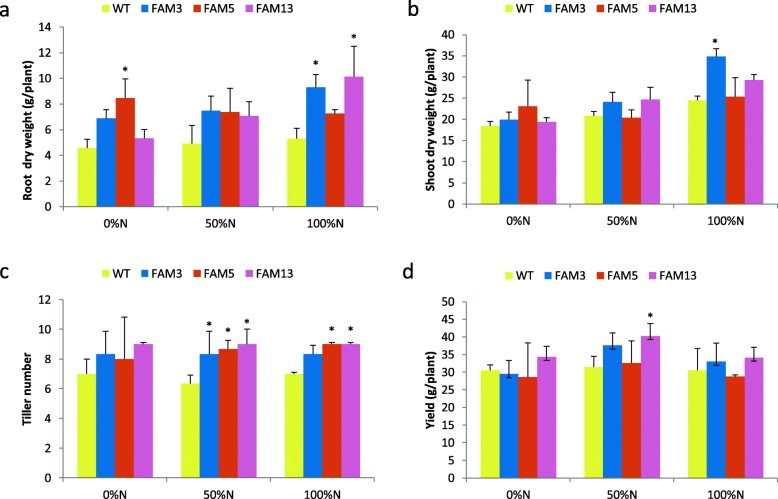
Greenhouse experiment for evaluation of nitrogen use efficiency in transgenic events and wild type. **a** Root dry weight of three transgenic events and wild type at 3 N doses. **b** Shoot dry weight of three transgenic events and wild type at 3 N doses. **c** Tiller number of three transgenic events and wild type at 3 N doses. **d** Grain yield of three transgenic events and wild type at 3 N doses. Bars represent the means ± s.d. of three replicates. Means labeled with * indicate significant differences from WT at the corresponding N dose at *P* < 0.05

**Fig. 3:**
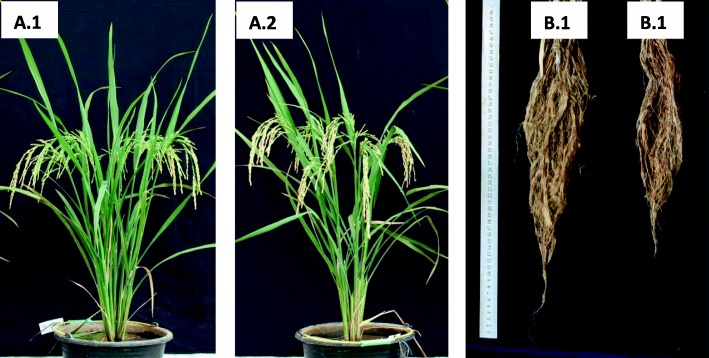
Performances of T1 transgenic rice events and WT at harvest stage at the low N condition in the greenhouse. A1 = shoot of event FAM13, A2 = shoot of wild type, B1 = root of event FAM13, B2 = root of wild type

Root dry weights of FAM 3 and FAM13 were significantly higher than WT at N 90 kg/ha, whereas the one in FAM5 was significantly higher than WT at N 0 kg/ha (Fig. 2a and Table 3). Transgenic plants showed longer and more dense root systems than control plants (Fig. 3). Shoot dry weight of FAM3 was significantly higher than WT at N 180 kg/ha (Fig. 2b and Table 3). Tiller numbers of FAM3 and FAM 13 were significantly higher than WT at N 90 kg/ha and N 180 kg/ha, whereas the one in FAM5 was significantly higher than WT only at N 90 kg/ha (Fig. 2c, Table 3, and Fig. 3). Grain yield of FAM13 significantly outperformed WT at N 90 kg/ha (Fig. 2d and Table 3).

All transgenic events showed higher total N contents than wild type (Table 4). Transgenic events at N 90 kg/ha showed higher grain NUE than at N 180 kg/ha (Table 4). Although in general the increase in N uptake efficiency of transgenic events at N 180 kg/ha was higher than at N 90 kg/ha, but the increase in grain yield of transgenic events at N 90 kg/ha was in general higher than at N 180 kg/ha (Table 4).

**Table 4:** Absorption, agronomic, and grain NUE values in *CsAlaAT2* transgenic events

Lines	Character	N fertilizer dosage	
		N 50%	N 100%
Wild type	Total biomass (gram/hill)	25.76 ± 1.99	29.83 ± 1.19
	Grain yield/plant (gram/hill)	31.48 ± 3.06	30.55 ± 6.18
	N content in plant	0.28 ± 0.1	0.27 ± 0.03
	Absorption NUE	0.284	0.14
	Agronomical NUE	25.76	14.91
	Grain NUE	31.48	15.28
FAM3	Total biomass (gram/hill)	31.6 ± 3.36	44.13 ± 1.36
	Grain yield/plant (gram/hill)	37.58 ± 3.57	33.02 ± 5.23
	N content in plant	0.39 ± 0.07	0.56 ± 0.13
	Absorption NUE	0.386	0.28
	Agronomical NUE	31.60	22.06
	Grain NUE	37.60	16
	Increase in N uptake efficiency (%)*	34.48	107.4
	Increase in grain yield (%)**	19.40	8
FAM5	Total Biomass (gram/hill)	30.91 ± 0.88	37.74 ± 1.33
	Grain yield/plant (gram/hill)	33.02 ± 3.42	30.63 ± 3.34
	N content in plant	0.43 ± 0.06	0.36 ± 0.12
	Absorption NUE	0.376	0.297
	Agronomical NUE	30.91	18.87
	Grain NUE	33	15.3
	Increase in N uptake efficiency (%)*	48.27	33.33
	Increase in grain yield (%)**	4.9	0.3
FAM13	Total Biomass (gram/hill)	31.78 ± 4	39.39 ± 1.45
	Grain yield/plant (gram/hill)	40.28 ± 3.53	34.09 ± 3.00
	N content in plant	0.32 ± 0.02	0.35 ± 0.07
	Absorption NUE	0.35	0.159
	Agronomical NUE	31.78	19.70
	Grain NUE	40.2	17
	Increase in N uptake efficiency (%)*	10.34	29.63
	Increase in grain yield (%)**	27.95	11.58

*Increase in N uptake efficiency is calculated from the difference between the value of the N content of transgenic event and control (wild-type) divided by the value of N content of control line multiplied by 100%

**Increased in the grain yield is calculated from the difference between the results of grain yield/plant of transgenic lines to control (wild-type) divided by grain yield/plant of control line multiplied by 100%

## Discussion

In our pre-screening experiment, among 13 events evaluated, only 2 events, i.e. FAM5 and FAM13, showed significantly higher root biomass than control plant. This is common for genetic engineering to improve specific trait in plant, as shown in Golden Rice 2 [24]. From 619 events developed, only 23 events showed high carotenoid content. Variation among events in the efficacy level is determined by several factors, such as copy number, positional effect, and integrity of T-DNA insert.

Our results indicate that expression of cucumber *AlaAT2* in transgenic rice plants under the control of a root-specific promoter (*OsAnt1*) increased root biomass, tiller number, and grain yield. In comparison with controls, transgenic rice plants showed significant increases in N content.

Correlation analysis among the traits showed that the only trait that had a positive correlation with grain yield was tiller number. On the other hand, root dry weight was the only trait that had a positive correlation with tiller number. In comparison with controls, transgenic plants showed longer and more dense root systems (Fig. 3), which may be beneficial to the transgenic events because root architecture system has been known to be closely related to the efficiency of nitrogen absorption and is a major determinant for the nitrogen use efficiency [19, 37].

Overexpression of *CsAlaAT2* in the root might trigger hypoxic-like response that would stimulate ethylene biosynthesis [1]. Ethylene has been known for its role in promoting root growth and development [3]. In addition, overexpression of *AlaAT* would increase the use and production of glutamate [1] that has been known for its role in modulating the mitotic activity in the apical meristem [18, 36] and the dynamic elongation of root cell files [29].

Root expression of *CsAlaAT2* would also produce alanine that is transported to the shoots for usage or storage. In contrast to glutamate, plants do not sense the transport of N as alanine, which leads to the formation of a positive feedback loop where the plant improves N uptake and in turn improves C metabolism [1].

We tested three transgenic events in the phenotypic evaluation in the greenhouse. In general, the transgenic events maintained higher tiller number as compared to WT irrespective of N levels (Fig. 2c). This improvement in tillering capacity might be due to increased N content in transgenic plants (Table 4). It has been shown previously that each node of rice culm contains a tiller primordium, and tiller formation depends largely on the stored nitrogen and carbohydrates in the culm [31].

Transgenic events showed up to 28% yield increase compared to WT when grown under limiting N50%, and lower yield increase (up to 12%) when grown under N100%. The capacity of transgenic plants to uptake nitrogen at N100% is not the limiting factor, because the transgenic events showed higher increase in N uptake efficiency when grown under N100% (29.63–107.4%) as compared to under N50% (4.9–34.48%). Instead, this may indicate that 90 kg/ha nitrogen (N50%) is an optimum dose to reach a good balance with the doses of phosphorus and potassium we used in this experiment. It has been shown previously in rice that relationship between N fertilizer rate and grain yields is not linear [38].

## Conclusions

In summary, we present here the development of transgenic rice plants overexpressing *CsAlaAT2* and assessment of the responses of these plants to different levels of supplied N. We identified a transgenic event that had a significantly higher grain yield than wild-type control under N 90 kg/ha. The increased yield was correlated with increased tiller number. Transgenic rice plants showed significant increases in nitrogen use efficiency. Our current findings suggest that the cucumber *AlaAT2* gene is efficacious to improve nitrogen use efficiency in transgenic rice. Using this gene for large-scale transformation should provide opportunity to identify transgenic rice events which are better in nitrogen use efficiency and, at the same time, have phenotype, nutrient composition, and molecular characteristics that comply with the biosafety regulatory requirements. Cultivating these highly efficacious and clean NUE events will have significant economic and environmental impacts in the rice-based agricultural system.

## Data Availability

The datasets generated during and/or analyzed during the current study are available from the corresponding author on reasonable request. The plant seeds generated during the current study have been deposited in the seed bank managed by Indonesian Center for Agricultural Biotechnology and Genetic Resources (ICABIOGRAD) and are available from the Director of ICABIOGRAD on reasonable request with appropriate material transfer agreement and comply with applicable national or international regulation.

## Abbreviations

agNUE: Agronomic nitrogen use efficiency
*AlaAT*: *Alanine aminotransferase*
ANOVA: Analysis of variance
aNUE: Absorption nitrogen use efficiency
C: Carbon
cDNA: Complementary DNA
*CsAlaAT*: *Cucumis sativus alanine aminotransferase*
*CsAlaAT*2-FL-F: *CsAlaAT2*-full length-forward
*CsAlaAT*2-FL-R: *CsAlaAT2*-full length-reverse
DAT: Days after transfer
DIG: Digoxigenin
DW: Dry weight (g)
GluA2: Glutelin A2
gNUE: Grain nitrogen use efficiency
GPP: Grains per panicle
GUS: Glucoronidase
GY: Grain yield (g/plant)
hpt-F: Hygromycin phosphotransferase-forward
hpt-R: Hygromycin phosphotransferase-reverse
MS: Murashige and Skoog
N: Nitrogen
Ns: Not significant
NUE: Nitrogen use efficiency
*OsAnt1*: *Oryza sativa Antiquitin 1*
PCR: Polymerase chain reaction
PH: Plant height
PL: Panicle length
RDW: Root dry weight (g)
RNA: Ribonucleic acid
RNC: Root N concentration (mg/g DW)
SDW: Shoot dry weight (g)
SNC: Shoot N concentration (mg/g DW)
T-DNA: Transfer-deoxyribonucleic acid
TN: Tiller number
WT: Wild type
